# Microbial Community Structure and Arsenic Biogeochemistry in an Acid Vapor-Formed Spring in Tengchong Geothermal Area, China

**DOI:** 10.1371/journal.pone.0146331

**Published:** 2016-01-13

**Authors:** Zhou Jiang, Ping Li, Dawei Jiang, Xinyue Dai, Rui Zhang, Yanhong Wang, Yanxin Wang

**Affiliations:** 1 State Key Laboratory of Biogeology and Environmental Geology, China University of Geosciences, Wuhan, People's Republic of China; 2 School of Environmental Studies, China University of Geosciences, Wuhan, People's Republic of China; The University of Akron, UNITED STATES

## Abstract

Arsenic biogeochemistry has been studied extensively in acid sulfate-chloride hot springs, but not in acid sulfate hot springs with low chloride. In this study, Zhenzhuquan in Tengchong geothermal area, a representative acid sulfate hot spring with low chloride, was chosen to study arsenic geochemistry and microbial community structure using Illumina MiSeq sequencing. Over 0.3 million 16S rRNA sequence reads were obtained from 6-paired parallel water and sediment samples along its outflow channel. Arsenic oxidation occurred in the Zhenxhuquan pool, with distinctly high ratios of arsenate to total dissolved arsenic (0.73–0.86). Coupled with iron and sulfur oxidation along the outflow channel, arsenic accumulated in downstream sediments with concentrations up to 16.44 g/kg and appeared to significantly constrain their microbial community diversity. These oxidations might be correlated with the appearance of some putative functional microbial populations, such as *Aquificae* and *Pseudomonas* (arsenic oxidation), *Sulfolobus* (sulfur and iron oxidation), *Metallosphaera* and *Acidicaldus* (iron oxidation). Temperature, total organic carbon and dissolved oxygen significantly shaped the microbial community structure of upstream and downstream samples. In the upstream outflow channel region, most microbial populations were microaerophilic/anaerobic thermophiles and hyperthermophiles, such as *Sulfolobus*, *Nocardia*, *Fervidicoccus*, *Delftia*, and *Ralstonia*. In the downstream region, aerobic heterotrophic mesophiles and thermophiles were identified, including *Ktedonobacteria*, *Acidicaldu*s, *Chthonomonas* and *Sphingobacteria*. A total of 72.41–95.91% unassigned-genus sequences were derived from the downstream high arsenic sediments 16S rRNA clone libraries. This study could enable us to achieve an integrated understanding on arsenic biogeochemistry in acid hot springs.

## Introduction

Acid hot springs provide unique environments for the evolution and establishment of microbial communities and their response to various biogeochemical and metabolic processes involving hydrogen (H_2_), sulfur (S), iron (Fe) and arsenic (As) [1–6]. Generally, all acid hot springs are classified into two different water types: acid sulfate-Cl water and sulfate water with low Cl [7, 8]. Acid sulfate-Cl hot springs are viewed to form by sulfide oxidation after saturated Na-Cl geothermal water is exposed to the Earth's surface, and thus have decreased pH values but retain high K, Na, F, Cl, Li, B and As concentrations as geothermal reservoir water [7]. The high As concentration of these environments has attracted attention of several groups studying As redox speciation [1, 9–11]. Results of previous studies illustrated that arsenite (As(III)) was predominant in the source water of acid sulfate-Cl hot springs, and was oxidized to arsenate (As(V)) once being discharged along the outlet by bacterial populations such as *Hydrogenobacter*, *Hydrogenobaculum*, *Sulfurihydrogenibium*, and *Thiomonas* [1, 9, 11, 12]. No As(III) oxidation in the source water of acid sulfate-Cl hot springs was observed because of sulfide inhibition by inactivating expressed As(III) oxidase enzyme (Aio) [4, 13]. Due to high concentrations of sulfide, Fe and As in those acid sulfate-Cl hot springs, elemental S and Fe depositions rich in As successively appeared along the outflow channel [9, 10, 14]. Various microbial populations such as *Sulfolobus*, *Sulfobacillus*, *Metallosphaera*, *Sufurihydrogenibium*, *Hydrogenobaculum*, *Thiomonas* and *Acidicaldus* had been found to respond to sulfide and Fe oxidations in those acid hot springs [1, 9, 14–16]. Comparatively, acid sulfate hot springs with low Cl have not studied systematically on As biogeochemistry. These geothermal features form by separation of the vapor phase rich in H_2_S from the reservoir and subsequent condensation and oxidation in shallow oxygen-rich groundwater or surface water [17].

Tengchong, located in southwestern of China, is a typical volcanic geothermal area and has abundant geothermal resources [18]. Zhenzhuquan in the Rehai geothermal field of Tengchong is a representative acid vapor-formed sulfate hot spring low in Cl, with 128.2 mg/L sulfate, 39.2 mg/L Cl and 71.1 μg/L As, as well as low concentrations of K, Na, Li and B [19]. This geochemistry is distinctly different from the previously studied acid sulfate-Cl hot springs (sulfate: 21.0–144.9 mg/L; Cl: 473.6–1907.0 mg/L; As: 1.8–5.3 mg/L) [1, 9–11]. The As concentrations in the sediments of Zhenzhuquan outflow channel occur at up to 16.44 g/kg, which substantially exceeded the terrestrial abundance of As (1.5–3 mg/kg) and could pose a potential environmental risk [20]. Previous studies indicated that different microbial communities inhabited geothermal environments with distinct geochemistry, including different media such as water and sediment at the same sites of hot springs [21, 22]. Though some microbial studies on As in mats or sediments along those acid sulfate-Cl hot springs outlet had been conducted by clone library and metagenome sequencing [1, 9, 10, 23], arsenic geochemistry and corresponding microbial communities in acid sulfate hot spring with low Cl have yet to be fully understood. Therefore, the objectives of this study were to: (1) investigate the As geochemistry and microbial community structures both in water and sediments along the outflow channel of a typical acid sulfate hot spring with low Cl; (2) evaluate the potential microbially-mediated As oxidation process; and (3) assess the environmental factors shaping the microbial community structures.

## Materials and Methods

### Site description

No specific permission was required for the described field studies because no animal or human subjects were involved in this research. The sampling locations are not privately owned or protected in any way. The field studies were not involved in endangered or protected species.

**Fig 1:**
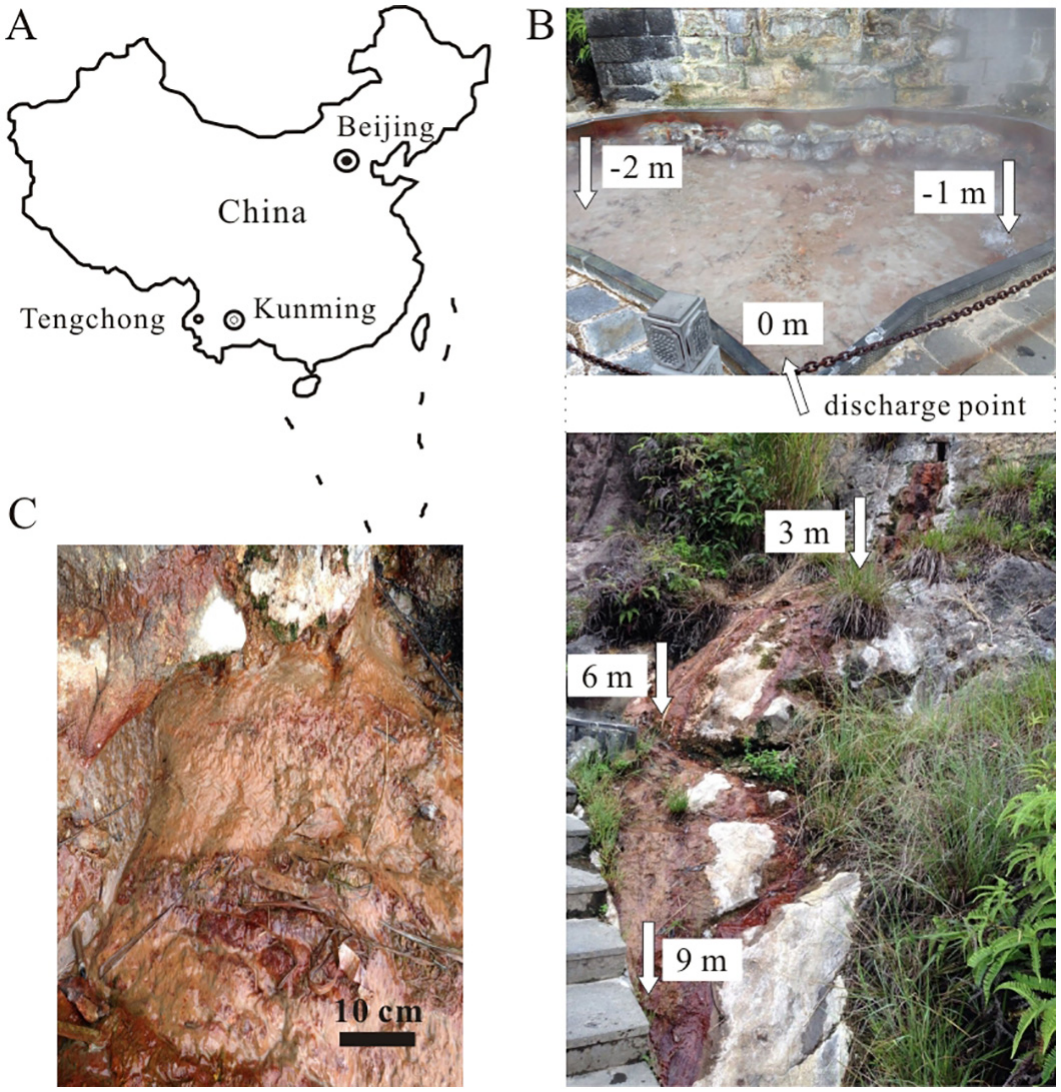
A map showing the location of Tengchong in China (A), Zhenzhuquan located in Rehai, Tengchong geothermal area and sampling sites distribution (B), and magnified photograph from sediment at the -6 m downstream (C).

As mentioned above, Zhenzhuquan (N24.9511°, E98.4361°) located in the Rehai geothermal field of Tengchong geothermal area in Yunnan, southwestern China, was selected for this study (Fig 1A). This spring is a heart-shaped acidic pool with a depth of 6–7 cm and a length of 4.36 m and is fed by numerous very small vigorous degassing vents (Fig 1B) [22]. It has a seasonally fluctuating temperature from 89.1 to 93.3°C and pH from 3.50 to 6.42, which results from the dilution of rainfall [17, 24]. Zhenzhuquan has a low discharge rate of 0.2 L/S and abundant reddish-brown sediments occur downstream from the source (Fig 1C). These sediments contain large amounts of As with concentrations up to 16.44 g/kg, even though As concentration is extremely low (48.16 μg/L) in the pool water [25]. Consequently, a transect of six sampling sites was established along Zhenzhuquan’s outflow channel and each sampling site was assigned a name according to the relative distance from commenced discharge point (0 m), such as 3 m, 6 m and 9 m downstream and -2 m and -1 m in the pool (Fig 1B). Parallel water and sediment samples at each site were collected in August, 2014.

### Field measurements and sample collection

Water temperature, pH and dissolved oxygen (DO) were measured in the field at the site of water collection using a hand-held meter. Concentrations of sulfide, ammonium, ferrous iron (Fe(II)) and total iron (Fe_Tot_) were also determined in the field with a Hach spectrophotometer (DR850, Hach Corp., USA) according to the manufacturer’s instructions. Water samples for laboratory measurements, e.g., anions, cations and dissolved organic carbon (DOC) were collected into 50 mL acid-washed polypropylene bottles and brown glass bottles respectively by filtration of spring water through 0.22 μm syringe polyethersulfone (PES) membrane filters (Pall Corp., NY, USA). Filtered biomass-containing membranes were placed into 15 mL sterile polypropylene tubes and immediately stored in dry ice. Water samples for cations and DOC were acidified with 1% v/v HNO_3_. Arsenic species separation was done on site, following the method reported by Le et al. [26]. Briefly, 10 mL of each water sample was passed through a silica-based strong anion-exchange cartridge (Supelco, USA) preconditioned with 50% methanol and deionized water before use. As(V) was retained in the cartridge and As(III) remained in the filtered solution. Subsequently, the cartridge was eluted with 10 mL 1 M HCl to release the bound As(V) to eluate samples. Sediment samples were collected in sterile 50 mL polypropylene tubes in duplicate by using sterile spoons and stored in ice. All samples for microbial community analysis (sediments and biomass-containing membranes) were stored in dry ice in the field and during transport, and then stored at -80°C in the laboratory until further analyses.

### Laboratory geochemical analysis

The cation and anion concentrations were measured by inductively coupled plasma-optical emission spectrometry (CAP6300, Thermo, USA) and ion chromatography (ICS1100, Dionex, USA), respectively. As_Tot_ and Fe_Tot_ in the sediments were extracted by 1:1 aqua regia digestion method in a water bath [27]. Pre-separated As(III) and As(V) from hot spring waters and extracted As_Tot_ from sediments were determined using liquid chromatography-hydride generation -atomic fluorescence spectrometry (LC-HG-AFS, Haiguang AFS-9780, Beijing) according to Jiang et al. [25]. Extracted Fe_Tot_ from sediments was determined by the 1,10-Phenanthroline-based assay: 10 mL extracted solutions was mixed with 5 mL acetate-sodium acetate buffer (pH = 4.6), 2.5 mL 1% hydroxylamine hydrochloride solution and 5 mL 0.1% 1,10-phenanthroline solution in 50 mL volumetric flask. The mixtures were made up to a volume of 50 mL with deionized water and allowed to stand for 10 min. The absorbance of each solution at 510 nm was measured with a spectrophotometer (UV1750, Shimadzu, Japan). Dissolved organic carton (DOC) of water samples and total organic carbon (TOC) of sediment samples were determined using a TOC analyzer (TOC-V_CPH_, Shimadzu, Japan) and a Macro elemental analyzer (Multi EA 4000, Analytik Jena, Germany), respectively.

### DNA extraction, amplification and sequencing

DNA was extracted from biomass-containing filters or from 0.5 g sediment samples using the FastDNA SPIN Kit for Soil (MP Biomedical, OH, USA). DNA concentrations were measured by Pico Green using a FLUOstar OPTIMA fluorescence plate reader (BMG LABTECH, Jena, Germany). The V4 region of 16S rRNA gene was amplified using the normal primer pair 515F (5’-GTGCCAGCMGCCGCGGTAA-3’) and 806R (5’-GGACTACHVGGGTWTCTAAT-3’) combined with Illumina adapter sequences, a pad and a linker of two bases, as well as barcodes on the reverse primers [28]. PCR amplification was carried out in a 25 μL reaction containing 2.5 μL 10× PCR buffer II (including dNTPs) (Invitrogen, Grand Island, NY), 0.4 μM of both forward and reverse primers, 10–15 ng DNA and 0.25 U high fidelity AccuPrime^™^ Taq DNA polymerase (Life Technologies) under the following program: initial denaturation at 94°C for 1 min, followed by 30 cycles of 94°C for 20 s, 53°C for 25 s, and 68°C for 45 s, and then a final extension at 68°C for 10 min. Reactions were performed in triplicate. Amplicons of each sample were combined and confirmed positive PCR products by agarose gel electrophoresis, and then quantified with PicoGreen. Finally, a total of 200 ng PCR product of each sample was pooled together and purified through QIAquick Gel Extraction Kit (Qiagen, Valencia, CA) and then was re-quantified with PicoGreen. Sample 16S rRNA clone libraries for sequencing were prepared according to the MiSeqTM Reagent Kit Preparation Guide (Illumina, San Diego, CA, USA) and the protocol described previously [29]. Briefly, sample denaturation was performed by mixing 10 μL of combined PCR products (2 nM) and 10 μL 0.2 M NaOH and incubated for 8 min at room temperature. Denatured DNA was diluted to 15 pM using HT1 buffer and mixed with a PhiX DNA library (final concentration 14.3%). A total of 600 μL sample mixture, together with customized sequencing primers for forward, reverse, and index reads, were loaded into the corresponding wells on the reagent cartridge of a 500-cycle v2 MiSeq kit. Sequencing was performed for 251, 12, and 251 cycles, respectively for forward, index, and reverse reads on a Illumina MiSeq system (Illumina, San Diego, CA).

### Sequence data preprocessing and statistical analysis

Raw sequences with perfect matches to barcodes were split to sample 16S rRNA clone libraries and were trimmed using Btrim with threshold of QC higher than 25 over 5 bp window size and the minimum length of 150 bp [30]. Forward and reverse reads with at least 50 bp overlap and lower than 5% mismatches were joined using Fast Length Adjustment of SHort reads (FLASH) [31]. After trimming of ambiguous bases (i.e. N), joined sequences with lengths between 247 and 258 bp were subjected to chimera removal by Uchime [32]. Operational taxonomic units (OTUs) clustering was through Uclust at 97% similarity level [33], and taxonomic assignment was through Ribosomal Database Project (RDP) classifier [34] with a minimal 50% confidence estimate. The above steps were performed through the Galaxy pipeline of Institute for Environmental Genomics in University of Oklahoma (http://zhoulab5.rccc.ou.edu/). Samples were rarefied at 12 955 sequences per sample. Singletons of generated OTU table were removed for downstream analyses.

All statistical analysis in this study was performed based on genus-level OTUs at the 97% similarity level under the Vegan package in R (http://www.r-project.org/), unless otherwise stated. A variety of alpha diversity indices were calculated including Chao1, Shannon and Equitability. Hierarchical cluster tree using unweighted pair group method with arithmetic means (UPGMA), principal coordinates analysis (PCoA) and non-metric dimensional scaling (NMDS) ordination were built to depict the community composition structure based on the Bray-Curtis dissimilarity matrix of detected OTUs. The Envfit function in the package of Vegan was used to overlay the significant environmental variables on the NMDS ordination. Analyses of similarity (ANOSIM), non-parametric multivariate ANOVA (ADONIS), multi-response permutation procedure (MRPP) and Mantel were performed to test for significant differences in microbial community compositions between sample types (i.e., water vs. sediment) and different locations (i.e., pool vs. downstream). The DNA sequences were deposited to the Short Read Archive database at NCBI (Accession number: SRP056673).

## Results

### Water and sediment geochemistry

**Fig 2:**
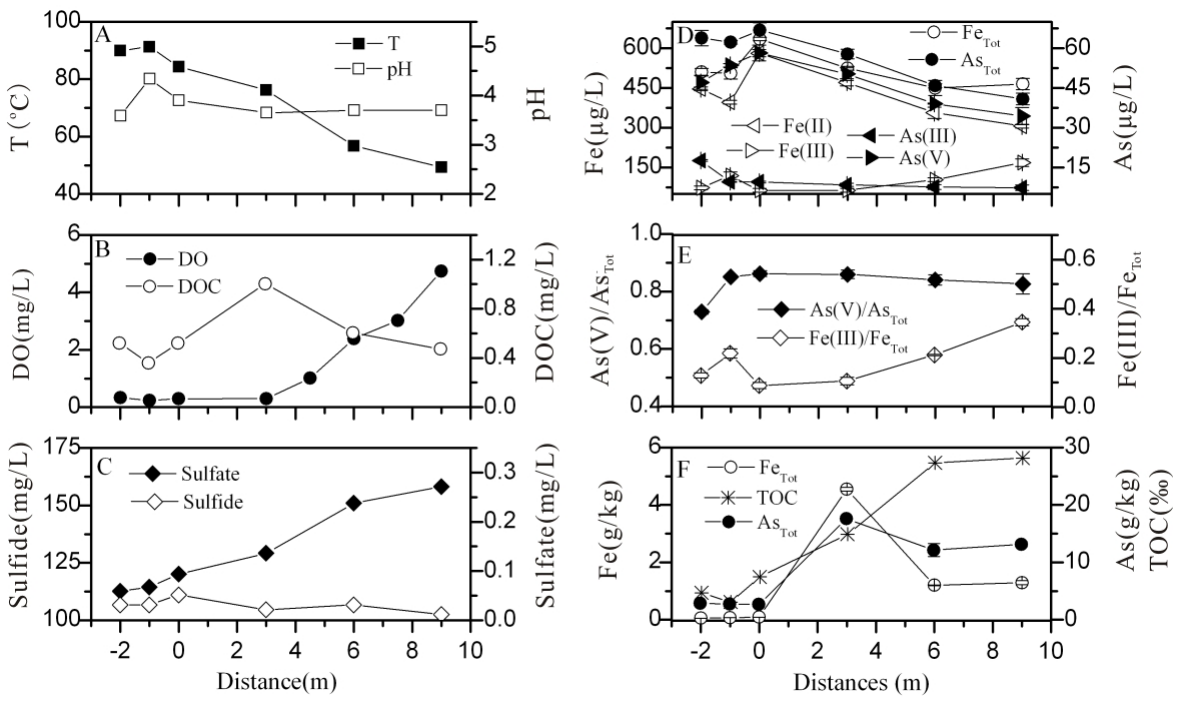
Distribution of selected geochemical data in water (A-E) and sediment (F) samples along the outflow channel of Zhenzhuquan. There were two more sampling sites (4.5 m and 7.5 m downstream) of DO. Error bars in D-F represented the standard deviations of duplicates.

Three water samples (-2 m, -1 m and 0 m) located in the pool of Zhenzhuquan showed similar physical and chemical conditions, with a slight fluctuation in temperature (84.3–91.3°C), pH (3.58–4.33), DO (0.23–0.33 mg/L), DOC (0.36–0.52 mg/L) and concentrations of various ions (Table 1 and S1 Fig), which suggested that Zhenzhuquan was derived by vigorous degassing from the same recharged source. The pool with a low DO average of 0.28 mg/L had correspondingly low ratios of Fe(III) and Fe_Tot_ (Fe(III)/Fe_Tot_: ~ 0.15), but contained high ratios of As(V) and As_Tot_ (As(V)/As_Tot_: ~ 0.81). Once the source water was discharged from the pool, most of physical-chemical parameters dramatically changed (Fig 2). Temperature ranged from 84.3°C to 49.3°C. The pH values showed a slight decline (pH = 3.89 at 0 m and pH = 3.69 at 12 m), which was consistent with acidic Succession Spring in Yellowstone National Park (YNP) [9]. The decrease in pH was primarily caused by evaporation, suggested by slight increase of F and Cl concentrations along the drainage (S1 Fig). Across sampling intervals, DO, sulfate and ratios of Fe(III) to Fe_Tot_ (Fe(III)/Fe_Tot_) significantly increased from 0.29 to 4.74 mg/L, 120.05 to 158.21 mg/L and 0.09 to 0.34, respectively. Dissolved Fe_Tot_ and As_Tot_ concentrations in water samples gradually declined from 0.64 to 0.45 mg/L and 66.83 to 44.83 μg/L respectively, coupled with significantly elevated concentrations of Fe_Tot_ (0.05–4.55 g/kg), As_Tot_ (10.53–16.44 g/kg) and TOC (1.37%-2.81%) in the downstream sediments.

**Table 1:** Geochemistry of water and sediment samples along Zhenzhuquan's outflow channel in Tengchong geothermal area.

Distance from discharge (m)	Aqueous phase																Solid phase		
	T°C	pH	mg/L									Fe(III)/Fe_Tot_	μg/L			As(V)/As_Tot_	g/kg		TOC (%)
			DO	DOC	Ammonia	Nitrate	Sulfide	Sulfate	Fe(II)	Fe(III)	Fe_Tot_		As(III)	As(V)	As_Tot_		Fe_Tot_	As_Tot_	
-2	89.9	3.58	0.33	0.52	3.00	bdl	0.03	112.66	0.45	0.07	0.51	0.13	17.29	46.55	63.84	0.73	0.05	0.50	0.27
-1	91.3	4.33	0.23	0.36	3.30	bdl	0.03	114.37	0.40	0.11	0.51	0.22	9.27	52.99	62.26	0.85	0.07	0.36	0.09
0	84.3	3.89	0.29	0.52	3.10	bdl	0.05	120.05	0.58	0.06	0.64	0.09	9.20	57.63	66.83	0.86	0.09	0.28	0.57
3	76.2	3.64	0.30	1.00	3.00	0.24	0.02	129.20	0.47	0.06	0.53	0.10	8.10	49.67	57.77	0.86	4.55	16.44	1.37
6	56.8	3.69	2.38	0.60	3.40	bdl	0.03	150.95	0.36	0.10	0.45	0.21	7.33	38.53	45.86	0.84	1.20	10.53	2.72
9	49.3	3.69	4.74	0.47	3.40	0.38	0.01	158.21	0.31	0.16	0.47	0.34	7.06	33.78	40.83	0.83	1.29	11.64	2.81

bdl:below detection limit (1 μg/L)

### Alpha diversity of microbial communities

**Fig 3:**
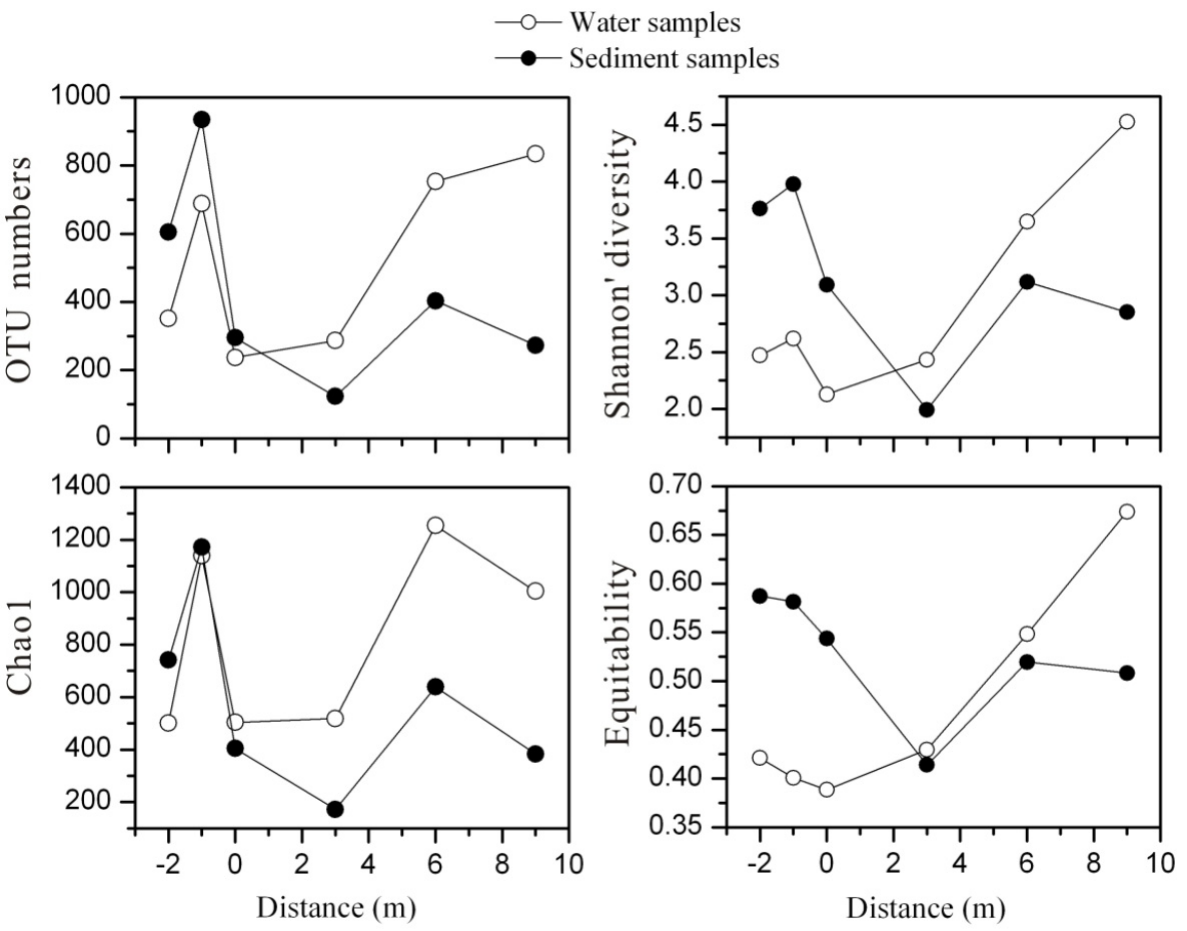
Alpha diversity indices distribution of microbial community structures of water and sediment samples along the outflow channel of Zhenzhuquan. The solid circles and open circles represented sediment and water samples respectively.

A total of 304 743 passing sequences were obtained from six-pair parallel water and sediment samples. After rarefaction at 12 955 sequences per sample, OTU clustering at 97% similarity level and removal of singletons, 155 050 sequences remained. A variety of taxa were present, with 123–834 observed and 172–1173 predicted OTUs (based on Chao1) and coverage values ranging from 47.04% to 83.08% (S1 Table). Along the outlet, microbial community richness, Shannon diversity and equitability from water samples significantly increased, whereas those indices of sediment samples had a decline with remarkable minimums at 3 m (Fig 3). Additionally, in the pool of Zhenzhuquan, richness, diversity and equitability in sediment samples were much higher than those in water samples. However, these indices were distinctly lower in sediment samples than those of water samples downstream. These alpha diversity differences between water and sediment samples can also be seen in their correlation with physical-chemical parameters: Shannon diversity and equitability of water samples were significantly correlated with DO, sulfate, Fe(III)/Fe_Tot_, Fe(II), dissolved As_Tot_, temperature and TOC, while these indices of sediment samples were significantly correlated with DOC, solid Fe_Tot_ and As_Tot_ in the sediments (Table 2).

**Table 2:** Correlation between alpha diversity indices at 97% similarity OTU level and environment factors.

Environment factors		Water samples			Sediment samples		
		Chao1	Shannon' diversity	Equitability	Chao1	Shannon' diversity	Equitability
Aqueous phase	DO	+0.492	+0.978***	+0.991***	-0.284	-0.201	-0.134
	Sulfate	+0.511	+0.911*	+0.935**	-0.456	-0.445	-0.404
	Fe(III)/Fe_Tot_	+0.742	+0.896*	+0.829*	+0.181	+0.127	+0.105
	Fe(II)	-0.756	-0.895*	-0.829*	-0.144	-0.045	+0.023
	As_Tot_	-0.655	-0.952**	-0.938**	+0.233	+0.277	+0.287
	T	-0.469	-0.938**	-0.939**	+0.486	+0.460	+0.411
	DOC	-0.448	-0.221	-0.122	-0.717	-0.857*	-0.896*
Solid phase	Fe_Tot_	-0.218	+0.031	+0.107	-0.679	-0.895*	-0.964**
	As_Tot_	+0.107	+0.480	+0.544	-0.694	-0.856*	-0.897*
	TOC	+0.461	+0.863*	+0.896*	-0.508	-0.504	-0.496

*p<0.05,

**p<0.01,

***p<0.001

### Microbial community compositions

**Fig 4:**
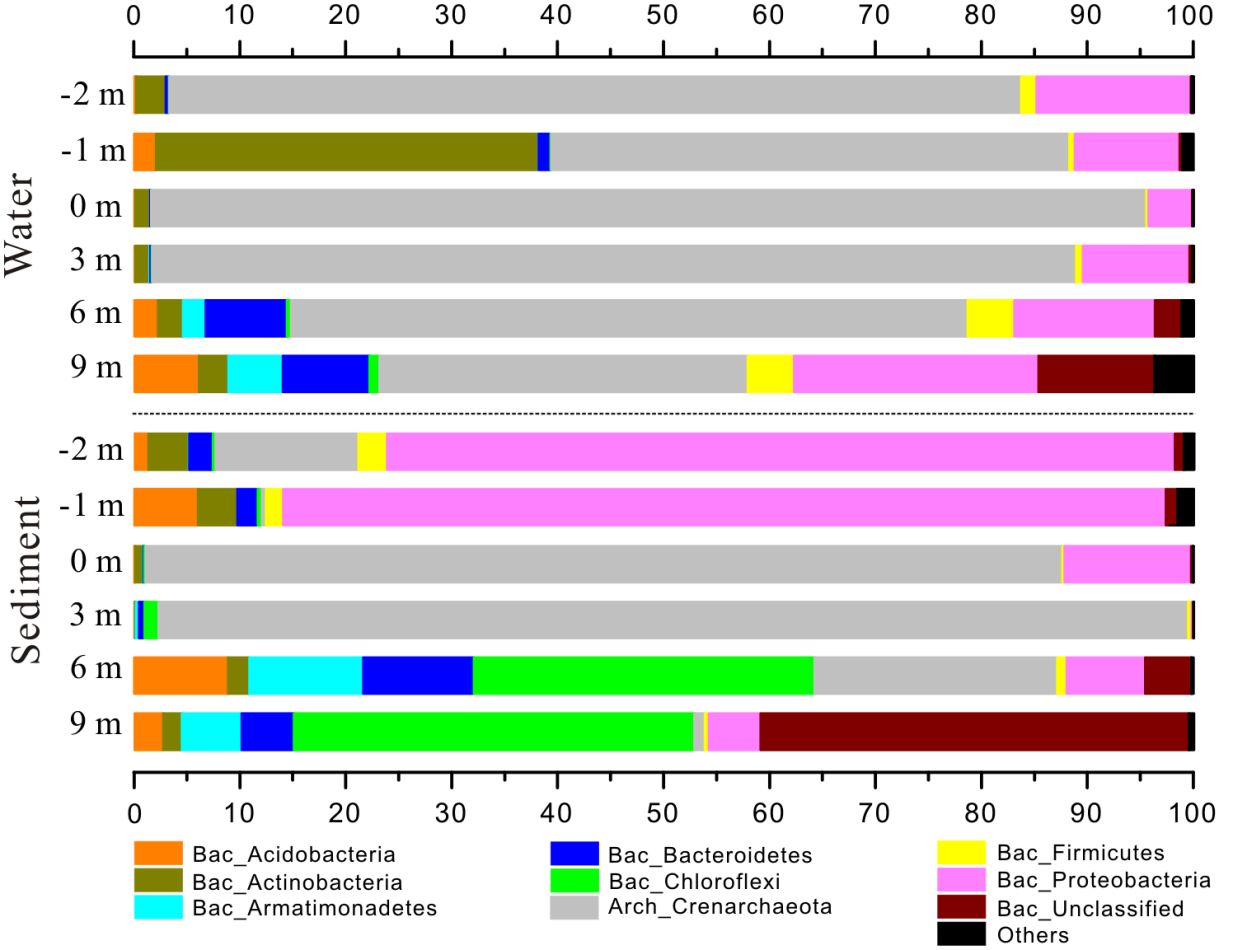
Microbial community structures distribution of water and sediment samples at phylum level.

Microbial community compositions were distinctly different between water and sediment samples (Fig 4). In the pool, water sample 16S rRNA clone libraries were dominated by *Crenarchaeota* (48.90 to 93.92%) with the rest mainly composed of *Actinobacteria* (1.42–36.14%) and *Proteobacteria* (4.12–14.57%), while sediment sample 16S rRNA clone libraries at -2 m and -1 m were predominated by *Proteobacteria* (74.36–83.31%). Along the outlet from 0 m to 9 m, *Crenarchaeota* dominated in water samples gradually declined from 93.92% to 34.79%, which was coupled with slight increase of *Acidobacteria* (0.07–6.06%), *Actinobacteria* (1.42–2.80%), *Armatimonadetes* (0.01–5.13%), *Bacteroidetes* (0.07–8.18%), *Firmicutes* (0.22–4.36%) and *Proteobacteria* (4.12–23.10%). However, initial dominant *Crenarchaeota* in sediment samples was generally substituted by *Chloroflexi* along the outflow channel, ranging from 0.13% to 37.76%. There were some unclassified phyla in downstream samples 16S rRNA clone libraries, especially the sediment at 9 m (Fig 4). Generally, *Acidobacteria*, *Armatimonadetes*, *Bacteroidetes* and *Chloroflexi* positively correlated with DO, ammonia, sulfate, Fe(III)/Fe_Tot_, dissolved As_Tot_ and TOC, and negatively correlated with temperature, aqueous Fe(II), solid Fe_Tot_ and As_Tot_ (S2 Table).

**Fig 5:**
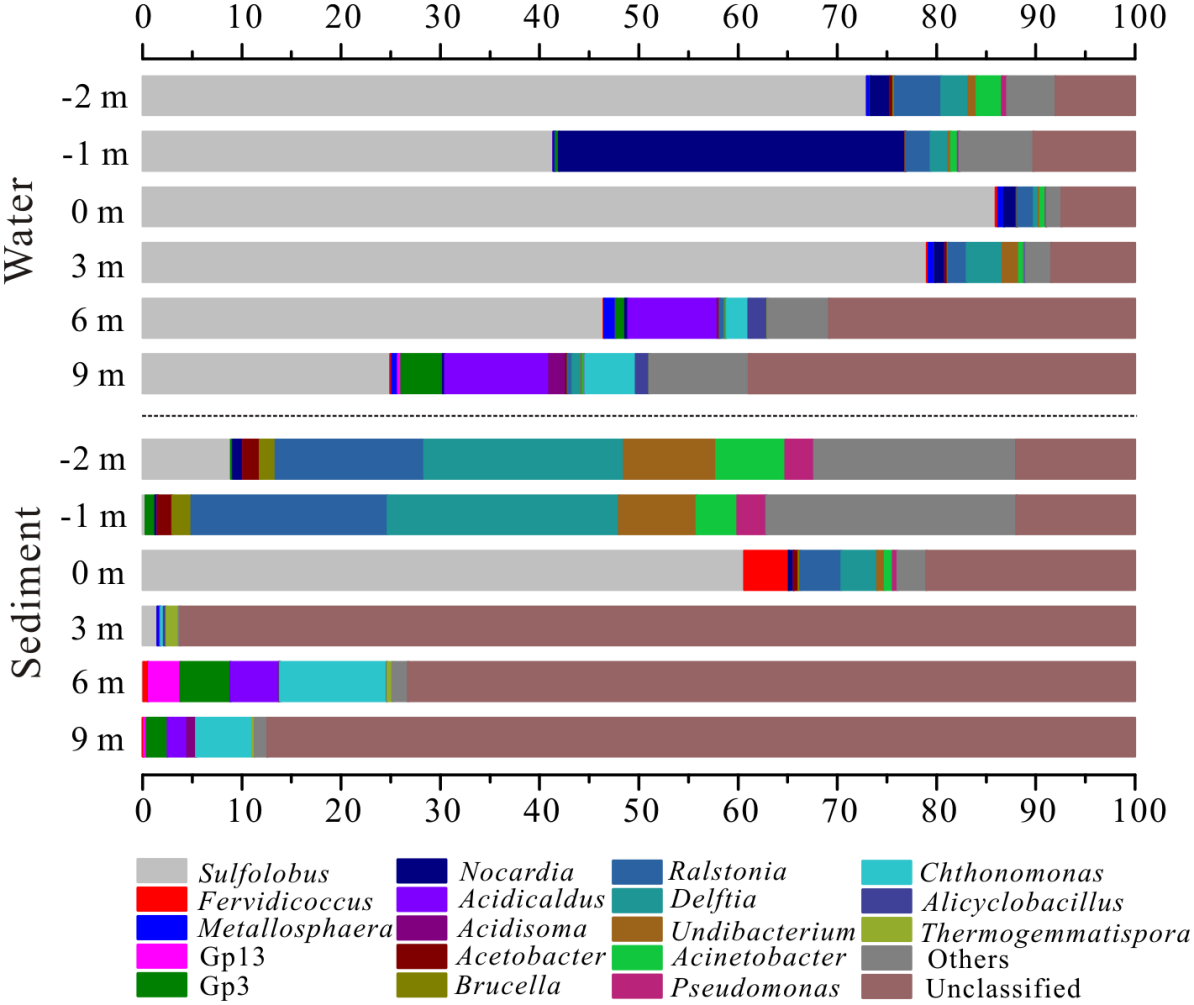
Microbial community structures distribution of water and sediment samples at genus level. The relative abundances of all genera in different samples were displayed in S3 Table.

At the genus level, water sample 16S rRNA clone libraries in the pool were composed of the *Crenarchaeota Sulfolobus* (41.38–85.95%), the *Actinobacteria Nocardia* (1.17–34.90%) and the *Proteobacteria Ralstonia* (1.66–4.66%), *Delftia* (0.51–2.76%) and *Acinetobacter* (0.58–2.57%) (Fig 5, S3 Table). In contrast, sediment sample 16S rRNA clone libraries in the pool (-2 m and -1 m) dominated by *Proteobacteria* correspondingly were harbored by more *Ralstonia* (14.96–19.76%), *Delftia* (20.13–23.29%), *Acinetobacter* (4.10–6.99%), *Undibacterium* (7.84–9.29%) and *Pseudomonas* (2.89–2.92%), and less *Sulfolobus* (0.30–8.89%). Along the outflow channel, *Sulfolobus* in water samples gradually decreased from 85.95% to 24.94%, coupled with elevated Gp3 (0.02–4.22%) of *Acidobacteria*, *Chthonomonas* (0–5.11%) of *Armatimonadetes*, *Alicyclobacillus* (0.02–1.93%) of *Firmicutes* and *Acidicaldus* (0–10.48%) of *Proteobacteria*. However, in downstream sediment samples, 73.25–96.21% sequences did not belong to any known genus except for minor Gp3 (0.01–5.05%), *Chthonomonas* (0.03–10.77%) and *Acidicaldus* (0.01–4.86%). Most of unknown-genus sequences were from class *Thermoprotei* (86.48–97.18%) of *Crenarchaeota* at 0 m and 3 m and class *Ktedonobacteria* (31.73–37.34%) of *Chloroflexi* at 6 m and 9 m (S2 Fig). Moreover, at the class level, *Thermoprotei* was predominant in all water samples and sediment samples at 0 m and 3 m, but replaced by *Ktedonobacteria* in sediment samples at 6 m and 9 m (S3 Fig). *Acidicaldus*, *Gp3*, *Acidisoma* and *Chthonomonas* positively correlated with DO, ammonia, sulfate, Fe(III)/Fe_Tot_, dissolved As_Tot_ and TOC, and negatively correlated with temperature, aqueous Fe(II) and As_Tot_ (S4 Table).

### Microbial community structure statistics

**Fig 6:**
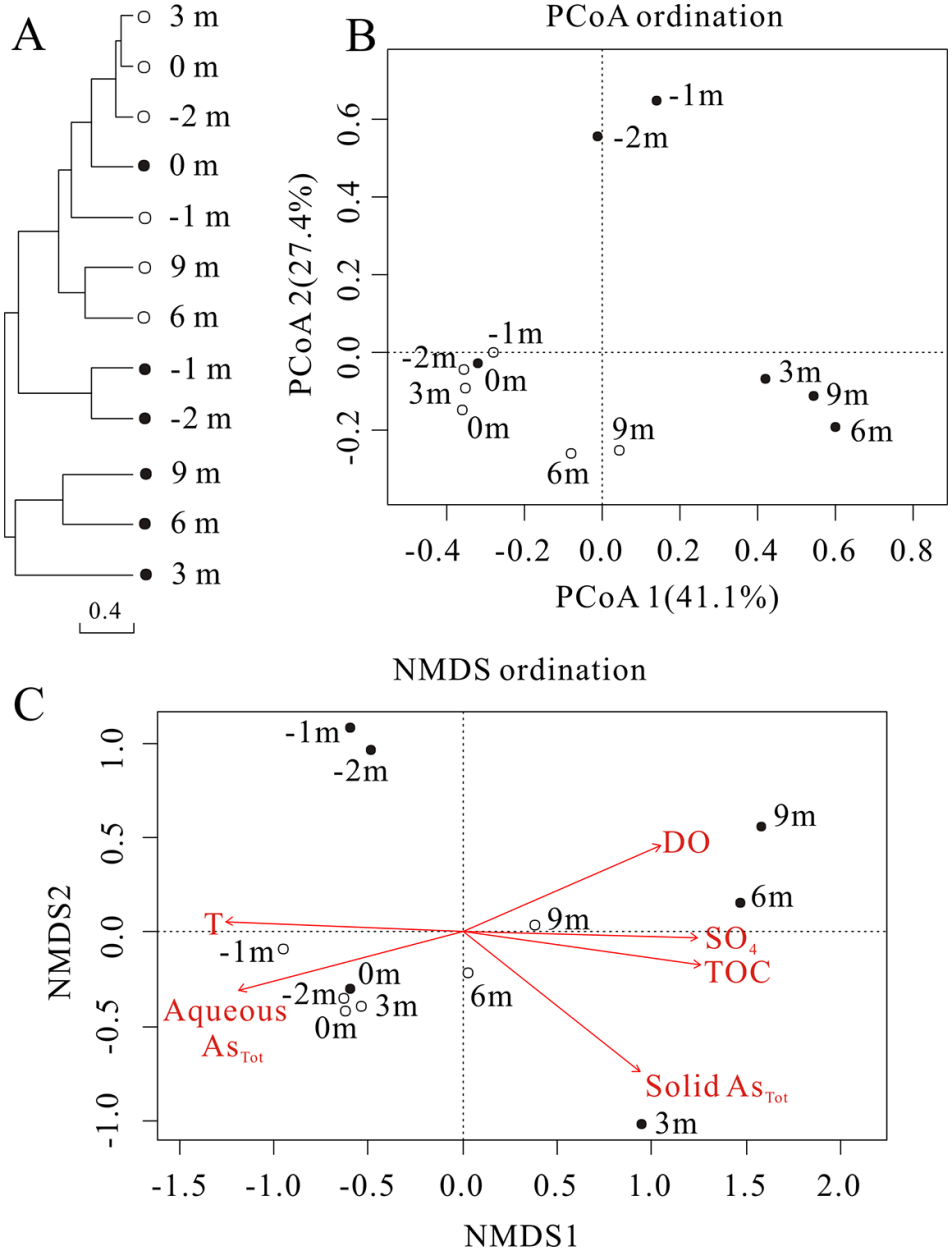
Microbial community distribution patterns at the 97% similarity OTU level. All analyses were performed based on Bray-Curtis dissimilarity of normalized OTU abundances of samples. The solid circles and open circles represented sediment and water samples respectively. A: The hierarchical cluster tree using unweighted pair group method with arithmetic means (UPGMA). B: Principal coordinates analysis (PCoA) scatter plot. The first two factors of PCoA1 and PCoA2 could explain 41.13% and 27.37% variations, respectively. C: Non-metric multidimensional scaling (NMDS) ordination plot. A biplot was overlaid on the ordination to identify environmental factors that were correlated with microbial community structure. The length of the line corresponds to the degree of the correlation. Only variables that had a significant correlation (P< 0.05) are depicted.

Based on Bray-Curtis dissimilarity at the 97% similarity OTU level, an UPGMA cluster tree of the microbial community populations showed that sediment samples were divided into two groups (pool and downstream), and distinctly separated from the water samples (Fig 6A). A similar result was also revealed by PCoA analysis with explained 68.5% of the observed variation (Fig 6B). Four complimentary non-parametric multivariate statistical tests including ADONIS, ANOSIM, MRPP and Mantel further confirmed the significant differences of microbial communities between not only pool and downstream samples, but also water and sediment samples (S5 Table). Results of Envfit function indicated that six geochemical parameters were significantly correlated (P<0.05) with microbial community structure along the outlet, including temperature, DO, sulfate, aqueous As_Tot_, solid TOC and As_Tot_ with R^2^ values of 0.63, 0.53, 0.62, 0.60, 0.65 and 0.57, respectively (Fig 6C). The similar directions of temperature and aqueous As_Tot_, and the opposite directions of DO, sulfate and TOC indicated correlations among these variables and did not necessarily suggest that all environmental factors were responsible driving forces of community structure. Significant environmental factors shaping microbial communities were selected according to general physiological niche of microbial populations.

## Discussion

### Potential As, S and Fe oxidation processes

In the Zhenzhuquan pool, the remarkably high As(V)/As_Tot_ ratio (0.73–0.86) suggested that As(III) oxidation occurred at the discharge source, which were distinctly different from previous studies that showed As(III) was predominate in the source water of acid sulfate-Cl hot springs, such as Dragon Spring (As(V)/As_Tot_ = 0.05) [11], Beowulf Spring (As(V)/As_Tot_ = 0.04) [10] and Succession Spring (As(V)/As_Tot_ = 0.04) [9] in YNP and Champagne Pool in New Zealand (As(V)/As_Tot_ = 0.04) [1]. The As oxidation in the pool might be mediated microbially. Previous studies demonstrated that sulfide was a potent inhibitor of microbially-mediated As(III) oxidation in acid systems by inactivating expressed As(III) oxidase enzyme (Aio) [2, 4, 13]. Consequently, different from acid sulfate-Cl hot springs with sulfide concentrations of 2.02–12.6 mg/L in those previous studies, the distinctly low concentrations of sulfide (0.03–0.05 mg/L) in the Zhenzhuquan pool allowed for microbial As(III) oxidation. Our previous study on *aioA* genes had demonstrated the presence of several groups of As(III)-oxidizing microorganisms in the pool, including a few unidentified families of *Aquificae* and some postulated archaea [25]. And in this study, based on 16S rRNA sequences, some microbial populations in sample 16S rRNA clone libraries were also highly similar with bacteria *Pseudomonas* and *Ralstonia* which were found to be capable of As oxidation in geothermal and other environments [35, 36].

Except for degassing in part, the lack of sulfide (0.03–0.05 mg/L) in the pool was primarily due to microbial sulfide oxidation, suggested by high sulfate concentrations (112.66–120.05 mg/L) and the presence of abundant *Sulfolobus* (41.38–85.95% in water sample libraries), a putative thermoacidophilic surfur-oxidizing archaeon (Table 1 and Fig 5) [22, 37, 38]. Concurrent with sulfide oxidation, Fe oxidation happened in the outflow channel, indicated by the decline of Fe(II) concentrations and increase of Fe(III)/Fe_Tot_ (Fig 2). Previous studies displayed that Fe(II) oxidation in acid hot springs was mediated by microorganisms, such as *Metallosphaera* str. MK1, *Sulfobacillus* str. MK2, *Sulfolobus* str. MK3, *Sulfolobales* str. MK5 and *Acidicaldu*s str. MK6 [9, 14]. In this study, some microbial populations belonging to *Thermoprotei* (such as *Sulfolobus*, and *Metallosphaera*) and *Acidicaldus* dominanated in downstream sample 16S rRNA clone libraries and were probably responsible for Fe(II) oxidation in the outflow channel (Fig 2) [14–16].

Due to lack of sulfide, yellow crystalline elemental S deposit presented in those acid sulfate-Cl hot springs did not appear downstream in this study [1, 9, 11]. Coupled Fe and As concentrations decline in water samples and increase in sediments suggested the co-deposition of As and Fe (Fig 2). However, extremely high As concentrations (up to 16.44 g/kg) and As/Fe mole ratios (2.70–6.72) in the sediments was significantly different from previous results from the acid sulfate-Cl hot springs (As/Fe mole ratios: 0.60 to 0.74) [9–11, 39–41]. Previous studies documented the clay minerals in geothermal areas, such as smectite and kaolinite, could host As concentrations up to 4 g/kg [8, 42–44]. Coincidentally, smectite and kaolinite were also detected in the downstream sediments of Zhenzhuquan by X-ray diffraction, which suggested that As might be also adsorbed on the clay mineral. Further investigation on mineralogy of the As-rich sediments downstream is warranted.

### Significant environmental factors shaping the microbial community structure

Generally, water and sediment sample 16S rRNA clone libraries along the outflow channel of Zhenzhuquan were dominated by *Thermoprotei* (mostly comprised of the genus *Sulfolobus*) (Fig 4 and S2 Fig), which was significantly different with sediments or waters from acid sulfate-Cl hot springs, mainly colonized by *Hydrogenobacter*, *Hydrogenobaculum* and *Sulfurihydrogenibium* of *Aquificae* [1, 9, 12]. This difference was probably derived by their distinct temperature and pH. *Hydrogenobacter* and *Sulfurihydrogenibium* favor circumneutral pH and *Hydrogenobaculum* has an optimal growth temperature of 60–70°C [45, 46], and microaerophilic acidophilic *Sulfolobus* prefer higher temperature of 65–85°C and more acidic pH of 2–3. These are all conditions similar to the Zhenzhuquan pool above at the 3 m site (DO: 0.23–0.33 mg/L; temperature: 76.2–91.3°C; pH: 3.58–4.33) (Table 1) [22]. This result was also consistent with distribution of *Sulfolobus* in acidic solfataras and hot springs in YNP [47, 48].

As revealed by non-parametric multivariate statistical analysis, there were significant differences in microbial community structures between not only pool and downstream samples, but also water and sediment samples along the outflow channel (S5 Table). Temperature, DO and TOC significantly shaped microbial community structure of upstream and downstream samples (Fig 6). Responding to the relatively low DO (0.23–0.30 mg/L) and high temperature (76.2–91.3°C) in upstream (Table 1), most microbial populations in 16S rRNA clone libraries were microaerophilic/anaerobic, thermophiles and hyperthermophiles, such as the dominant class *Thermoprotei* (48.90–97.18%, mainly comprised of *Sulfolobus* in water samples) [49], *Nocardia* (34.90%) in the water sample at -1 m [50], *Fervidicoccus* (4.46%) in sediment sample at 0 m [51], *Delftia* (20.13–23.29%) and *Ralstonia* (14.96–19.76%) in sediment samples at -1 m and -2 m [52] (Fig 5 and S2 Fig). With a decline in temperature (56.8–49.3°C) and increased DO (2.38–4.74 mg/L) as well as TOC (2.72–2.81%), more aerobic heterotrophic mesophiles and thermophiles correspondingly inhabited downstream samples libraries, such as *Ktedonobacteria* (31.72–37.34% in sediment samples) [53], *Acidobacteria* Gp3 (0.91–5.05%) [54], *Acidicaldu*s (1.98–10.48%) [55], *Chthonomonas* (2.12–10.16%) [56] and *Sphingobacteria* (4.94–10.40%) [57]. A significant positive correlation between sulfate and TOC (r = 0.989, p<0.001) along the outlet (Fig 2) implied that sulfide oxidation might be coupled to carbon fixation by facultatively chemoautotrophic archaea, such as above *Sulfolobus* and unclassified genera within the *Thermoprotei*, leading to TOC accumulation in downstream sediments [23]. Previous studies demonstrated that microbial community diversity increased with temperature decline and TOC or DOC increase in geothermal environments [21, 22]. However, in this study, the remarkably high As concentrations (up to 16.4 g/kg) and abnormal low alpha diversity in the downstream sediments (much lower than those of water at the same site) suggested that solid As_Tot_ in the sediments probably played a significant role in shaping microbial community structure (Fig 3) [58, 59]. Otherwise, it should be noted that even at the cutoff of 0.07, most sequences from the sediments 16S rRNA clone libraries (72.41–95.91%) could still not be assigned to a known genus, which suggested that some novel species possibly inhabit the high As sediments downstream (Fig 5 and S2 Fig).

## Conclusions

Arsenic oxidation mainly occurred in acid sulfate Zhenzhuquan pool with low chloride. Coupled with iron and sulfur oxidation along the outflow channel, arsenic was substantially accumulated in downstream sediments and appeared to significantly constrain their microbial community diversity. Temperature, total organic carbon and dissolved oxygen significantly shaped the different microbial communities between upstream and downstream samples of Zhenzhuquan. Some putative functional microbial populations were possibly related to arsenic oxidation (*Aquificae* and *Pseudomonas*), sulfur oxidation (*Sulfolobus*) and iron oxidation (*Sulfolobus*, *Metallosphaera* and *Acidicaldus*). A total of 72.41–95.91% unassigned-genus sequences in downstream high arsenic sediment 16S rRNA clone libraries probably implied the presence of some novel genera.

## Supporting Information

S1 Table Distribution of alpha diversity indices at the 97% similarity OTU level by re-sampling 12 955 reads in each sample. (DOC)

S2 Table Correlation between phylum at 97% similarity OTU level and environment factors. Only phylum significantly correlated with environment factors were displayed.
(DOC)

S3 Table The relative abundances of all genera in different samples. The unit is %.
(DOC)

S4 Table Correlation between genera at 97% similarity OTU level and environment factors. Only genera significantly correlated with environment factors were displayed.
(DOC)

S5 Table Significance tests of microbial community structures between different groups with four different statistical approaches. (DOC)

S1 Fig Normalized main cations and anions concentrations (divided by the sum) variation along the outflow channel of Zhenzhuquan. The values in parenthesis were averaged concentrations of ions with a unit of mg/L.
(TIF)

S2 Fig Distribution of microbial community compositions from water sediment samples at the class level. The ratios which exceeded 0.5% were displayed in this figure.
(TIF)

S3 Fig Change of main microbial community compositions at class level from water samples (A) and sediment samples (B) along the outflow channel of Zhenzhuquan. (TIF)
