# Supplementary material for: Microbial Community Structure and Arsenic Biogeochemistry in an Acid Vapor-Formed Spring in Tengchong Geothermal Area, China
Source: PLoS One. 2016 Jan 13;11(1):e0146331. doi: 10.1371/journal.pone.0146331 (PMC4711897; doi:10.1371/journal.pone.0146331)
Supplement: S1 Table — (DOC) [file pone.0146331.s001.doc]

**S1 Table. Distribution of alpha diversity indices at the 97% similarity OTU level** **by re-sampling 12 955 reads in each sample.**

| Samples | Distances | Reads numbers before re-sampling | Observed OTUs | Coverage^a^ of observed OTUs (%) | Chao1 | Shannon' diversity | Equitability |
| --- | --- | --- | --- | --- | --- | --- | --- |
| Water | -2 m | 33002 | 352 | 70.22 | 501 | 2.47 | 0.42 |
|  | -1 m | 21441 | 688 | 60.42 | 1139 | 2.62 | 0.40 |
|  | 0 m | 33607 | 237 | 47.04 | 504 | 2.12 | 0.39 |
|  | 3 m | 34677 | 287 | 55.31 | 519 | 2.43 | 0.43 |
|  | 6 m | 35446 | 753 | 60.05 | 1254 | 3.63 | 0.55 |
|  | 9 m | 16815 | 834 | 83.08 | 1004 | 4.53 | 0.67 |
| Sediment | -2 m | 14151 | 605 | 81.57 | 742 | 3.76 | 0.59 |
|  | -1 m | 13273 | 935 | 79.73 | 1173 | 3.98 | 0.58 |
|  | 0 m | 39313 | 295 | 72.96 | 404 | 3.09 | 0.54 |
|  | 3 m | 14903 | 123 | 71.39 | 172 | 1.99 | 0.41 |
|  | 6 m | 35160 | 403 | 63.05 | 639 | 3.12 | 0.52 |
|  | 9 m | 12955 | 273 | 71.23 | 383 | 2.85 | 0.51 |

^a^Coverage is the ratio of the observed OTUs to Chao1.
