# Supplementary material for: Microbial Community Structure and Arsenic Biogeochemistry in an Acid Vapor-Formed Spring in Tengchong Geothermal Area, China
Source: PLoS One. 2016 Jan 13;11(1):e0146331. doi: 10.1371/journal.pone.0146331 (PMC4711897; doi:10.1371/journal.pone.0146331)
Supplement: S2 Table — Only phylum significantly correlated with environment factors were displayed. (DOC) [file pone.0146331.s002.doc]

**S2 Table. Correlation between phylum at 97% similarity OTU level and environment factors.** Only phylum significantly correlated with environment factors were displayed.

| Environment factors | | *Acidobacteria* | *Actinobacteria* | *Armatimonadetes* | *Bacteroidetes* | *Chloroflexi* | *Crenarchaeota* | Unclassified | Others |
| --- | --- | --- | --- | --- | --- | --- | --- | --- | --- |
| Aqueous phase | T | -0.477 | +0.324 | -0.784** | -0.820** | -0.615* | +0.267 | -0.645* | -0.338 |
|  | pH | +0.191 | +0.693* | -0.272 | -0.242 | -0.205 | -0.216 | -0.181 | +0.150 |
|  | DO | +0.515 | -0.192 | +0.729** | +0.769** | +0.611* | -0.478 | +0.787** | +0.527 |
|  | DOC | -0.365 | -0.392 | -0.093 | -0.182 | -0.097 | +0.571 | -0.211 | -0.408 |
|  | Ammonia | +0.732** | +0.141 | +0.701* | +0.811** | +0.535 | -0.559 | +0.515 | +0.487 |
|  | Nitrate | +0.054 | -0.234 | +0.277 | +0.191 | +0.312 | -0.107 | +0.654* | +0.339 |
|  | Sulfide | -0.298 | +0.029 | -0.370 | -0.389 | -0.350 | +0.402 | -0.574 | -0.446 |
|  | Sulfate | +0.502 | -0.292 | +0.788** | +0.827*** | +0.617* | -0.282 | +0.642* | +0.349 |
|  | Fe(II) | -0.680* | -0.112 | -0.664* | -0.79** | -0.526 | +0.671* | -0.572 | -0.557 |
|  | Fe_Tot_ | -0.618* | +0.002 | -0.674* | -0.81** | -0.502 | +0.542 | -0.431 | -0.403 |
|  | As(III) | -0.346 | -0.038 | -0.380 | -0.357 | -0.299 | +0.016 | -0.308 | -0.174 |
|  | As_Tot_ | -0.615* | +0.151 | -0.798** | -0.884*** | -0.618* | +0.451 | -0.619* | -0.432 |
|  | Fe(III)/Fe_Tot_ | +0.649* | +0.202 | +0.576* | +0.657* | +0.501 | -0.707* | +0.707* | +0.671* |
|  | As(V)/As_Tot_ | +0.034 | +0.098 | -0.011 | -0.083 | -0.018 | +0.296 | -0.063 | -0.111 |
| Solid phase | Fe_Tot_ | -0.186 | -0.279 | +0.029 | -0.051 | +0.025 | +0.374 | +0.011 | -0.190 |
|  | As_Tot_ | +0.128 | -0.338 | +0.417 | +0.389 | +0.325 | +0.108 | +0.315 | +0.040 |
|  | TOC | +0.465 | -0.347 | +0.785** | +0.827*** | +0.601* | -0.214 | +0.573 | +0.273 |
| *p<0.05, **p<0.01, ***p<0.001 | | | | | | | | | |
