# Supplementary material for: Microbial Community Structure and Arsenic Biogeochemistry in an Acid Vapor-Formed Spring in Tengchong Geothermal Area, China
Source: PLoS One. 2016 Jan 13;11(1):e0146331. doi: 10.1371/journal.pone.0146331 (PMC4711897; doi:10.1371/journal.pone.0146331)
Supplement: S3 Table — The unit is %. (DOC) [file pone.0146331.s003.doc]

**S3 Table. The relative abundances of all genera in different samples. The unit is %.**

| Genus | Water | | | | | | Sediment | | | | | |
| --- | --- | --- | --- | --- | --- | --- | --- | --- | --- | --- | --- | --- |
|  | -2 m | -1 m | 0 m | 3 m | 6 m | 9 m | -2 m | -1 m | 0 m | 3 m | 6 m | 9 m |
| Unclassified genus | 8.02 | 10.24 | 7.40 | 8.44 | 30.83 | 38.92 | 11.98 | 11.95 | 21.03 | 96.21 | 73.25 | 87.40 |
| *Others* | 4.87 | 7.43 | 1.51 | 2.57 | 6.16 | 9.97 | 20.33 | 25.19 | 2.89 | 0.14 | 1.59 | 1.31 |
| *Thermogemmatispora* | 0.00 | 0.00 | 0.00 | 0.00 | 0.05 | 0.04 | 0.00 | 0.00 | 0.00 | 1.26 | 0.52 | 0.26 |
| Gp13 | 0.00 | 0.00 | 0.00 | 0.01 | 0.04 | 0.35 | 0.00 | 0.00 | 0.00 | 0.00 | 3.23 | 0.33 |
| Gp3 | 0.05 | 0.31 | 0.02 | 0.02 | 0.91 | 4.22 | 0.24 | 1.00 | 0.02 | 0.01 | 5.05 | 2.07 |
| *Nocardia* | 1.93 | 34.90 | 1.17 | 1.00 | 0.31 | 0.20 | 0.96 | 0.14 | 0.47 | 0.00 | 0.01 | 0.00 |
| *Acidicaldus* | 0.00 | 0.00 | 0.00 | 0.05 | 8.98 | 10.48 | 0.00 | 0.00 | 0.01 | 0.07 | 4.86 | 1.98 |
| *Acidisoma* | 0.00 | 0.02 | 0.00 | 0.01 | 0.25 | 1.73 | 0.06 | 0.12 | 0.01 | 0.00 | 0.09 | 0.90 |
| *Acetobacter* | 0.35 | 0.03 | 0.12 | 0.21 | 0.02 | 0.15 | 1.66 | 1.46 | 0.47 | 0.00 | 0.00 | 0.00 |
| *Brucella* | 0.15 | 0.02 | 0.01 | 0.10 | 0.01 | 0.01 | 1.59 | 1.92 | 0.15 | 0.00 | 0.00 | 0.00 |
| *Ralstonia* | 4.66 | 2.50 | 1.66 | 1.84 | 0.47 | 0.48 | 14.96 | 19.76 | 4.15 | 0.00 | 0.02 | 0.00 |
| *Delftia* | 2.76 | 1.79 | 0.51 | 3.58 | 0.09 | 0.88 | 20.13 | 23.29 | 3.59 | 0.00 | 0.02 | 0.00 |
| *Undibacterium* | 0.81 | 0.24 | 0.14 | 1.65 | 0.05 | 0.19 | 9.29 | 7.84 | 0.78 | 0.00 | 0.01 | 0.01 |
| *Acinetobacter* | 2.57 | 0.71 | 0.58 | 0.52 | 0.11 | 0.15 | 6.99 | 4.10 | 0.83 | 0.00 | 0.00 | 0.00 |
| *Pseudomonas* | 0.51 | 0.14 | 0.03 | 0.09 | 0.00 | 0.03 | 2.89 | 2.92 | 0.44 | 0.00 | 0.00 | 0.00 |
| *Chthonomonas/Armatimonadetes_*gp3 | 0.00 | 0.02 | 0.00 | 0.06 | 2.12 | 5.11 | 0.02 | 0.00 | 0.03 | 0.30 | 10.77 | 5.64 |
| *Alicyclobacillus* | 0.00 | 0.01 | 0.04 | 0.02 | 1.93 | 1.38 | 0.00 | 0.01 | 0.00 | 0.25 | 0.01 | 0.00 |
| *Fervidicoccus* | 0.01 | 0.01 | 0.26 | 0.17 | 0.08 | 0.20 | 0.00 | 0.00 | 4.46 | 0.01 | 0.47 | 0.11 |
| *Sulfolobus* | 72.98 | 41.38 | 85.95 | 79.01 | 46.43 | 24.94 | 8.89 | 0.30 | 60.61 | 1.50 | 0.10 | 0.01 |
| *Metallosphaera* | 0.34 | 0.24 | 0.60 | 0.64 | 1.15 | 0.56 | 0.00 | 0.00 | 0.06 | 0.25 | 0.01 | 0.00 |
