# Supplementary material for: Microbial Community Structure and Arsenic Biogeochemistry in an Acid Vapor-Formed Spring in Tengchong Geothermal Area, China
Source: PLoS One. 2016 Jan 13;11(1):e0146331. doi: 10.1371/journal.pone.0146331 (PMC4711897; doi:10.1371/journal.pone.0146331)
Supplement: S4 Table — Only genera significantly correlated with environment factors were displayed. (DOC) [file pone.0146331.s004.doc]

**S4 Table. Correlation between genera at 97% similarity OTU level and environment factors.** Only genera significantly correlated with environment factors were displayed.

| Environment factors | | Unclassified | *Thermogemmatispora* | *Gp3* | *Acidicaldus* | *Acidisoma* | *Ralstonia* | *Acinetobacter* | *Chthonomonas* | *Alicyclobacillus* | *Sulfolobus* |
| --- | --- | --- | --- | --- | --- | --- | --- | --- | --- | --- | --- |
| Aqueous phase | T | -0.642* | -0.227 | -0.738** | -0.794** | -0.710** | +0.595* | +0.586* | -0.784** | -0.571 | +0.326 |
|  | pH | -0.347 | -0.278 | -0.150 | -0.270 | -0.180 | +0.397 | +0.064 | -0.272 | -0.216 | -0.050 |
|  | DO | +0.538 | +0.030 | +0.747** | +0.753** | +0.877*** | -0.421 | -0.407 | +0.729** | +0.498 | -0.384 |
|  | DOC | +0.326 | +0.587* | -0.209 | -0.113 | -0.252 | -0.377 | -0.297 | -0.093 | -0.000 | +0.126 |
|  | Ammonia | +0.325 | -0.108 | +0.735** | +0.713** | +0.581* | -0.242 | -0.418 | +0.701* | +0.489 | -0.390 |
|  | Nitrate | +0.542 | +0.305 | +0.311 | +0.302 | +0.728** | -0.411 | -0.408 | +0.277 | +0.186 | -0.252 |
|  | Sulfide | -0.507 | -0.290 | -0.418 | -0.386 | -0.644* | +0.196 | +0.162 | -0.369 | -0.259 | +0.496 |
|  | Sulfate | +0.640* | +0.223 | +0.747** | +0.798** | +0.708** | -0.588* | -0.601* | +0.788** | +0.574 | -0.338 |
|  | Fe(II) | -0.446 | -0.082 | -0.706* | -0.675* | -0.645* | +0.153 | +0.182 | -0.663* | -0.467 | +0.595* |
|  | Fe_Tot_ | -0.480 | -0.197 | -0.666* | -0.675* | -0.482 | +0.207 | +0.171 | -0.674* | -0.497 | +0.555 |
|  | As(III) | -0.449 | -0.276 | -0.383 | -0.383 | -0.344 | +0.463 | +0.762** | -0.381 | -0.299 | +0.181 |
|  | As_Tot_ | -0.607* | -0.202 | -0.779** | -0.807** | -0.687* | +0.448 | +0.460 | -0.798** | -0.580* | +0.472 |
|  | Fe(III)/Fe_Tot_ | +0.360 | -0.081 | +0.674* | +0.602* | +0.801** | -0.118 | -0.236 | +0.576 | +0.376 | -0.529 |
|  | As(V)/As_Tot_ | +0.173 | +0.234 | -0.017 | -0.018 | -0.070 | -0.303 | -0.634* | -0.010 | +0.026 | +0.090 |
| Solid phase | Fe_Tot_ | +0.463 | +0.619* | -0.042 | +0.018 | +0.000 | -0.414 | -0.416 | +0.030 | +0.079 | -0.058 |
|  | As_Tot_ | +0.658* | +0.574 | +0.344 | +0.413 | +0.339 | -0.571 | -0.566 | +0.418 | +0.348 | -0.234 |
|  | TOC | +0.655* | +0.280 | +0.720** | +0.790** | +0.628* | -0.613* | -0.596* | +0.785** | +0.583* | -0.315 |
| *p<0.05, **p<0.01, ***p<0.001 | | | | | | | | | | | |
