# Supplementary material for: Microbial Community Structure and Arsenic Biogeochemistry in an Acid Vapor-Formed Spring in Tengchong Geothermal Area, China
Source: PLoS One. 2016 Jan 13;11(1):e0146331. doi: 10.1371/journal.pone.0146331 (PMC4711897; doi:10.1371/journal.pone.0146331)
Supplement: S5 Table — (DOC) [file pone.0146331.s005.doc]

**S5 Table. Significance tests of microbial community structures between different groups with four different statistical approaches.**

| Data sets | ADONIS^a^ | | ANOSIM^b^ | | MRPP^c^ | | Mantel^d^ | |
| --- | --- | --- | --- | --- | --- | --- | --- | --- |
|  | *F* | *P* | *R* | *P* | δ | *P* | *R* | *P* |
| Water vs Sediment | 3.0633 | 0.018 | 0.2852 | 0.023 | 0.6722 | 0.027 | 0.2354 | 0.165 |
| Pool vs Downstream^§^ | 3.0798 | 0.014 | 0.3815 | 0.019 | 0.6903 | 0.037 | 0.1322 | 0.314 |

All four tests are non-parametric multivariate analyses based on Bray-Curtis dissimilarities among samples. ^§^Pool: samples from -2m, -1m and 0m, downstream: samples from 3m, 6m and 9m. ^a^ ADONIS: Permutational multivariate analysis of variance using distance matrices. Significance tests were carried out using F-tests based on sequential sums of squares from permutations of the raw data. ^b^ ASNOSIM: Analysis of similarities which provides a way to test statistically whether there is a significant difference between two or more groups of sampling units. Statistic R is based on the difference of mean ranks between groups and within groups. The significance of observed R is assessed by permuting the grouping vector to obtain the empirical distribution of R under the null model. ^c^ MRPP: Multi-response permutation procedure. Statistic delta is the overall weighted mean of within-group means of the pairwise dissimilarities among sampling units. The significance test is the fraction of permuted delta that is less than the observed delta. ^d^ Mantel: Mantel statistic displays a matrix correlation between two dissimilarity matrices. Statistic R is based on pearson correlation method.
