# Supplementary figures and images for: Microbial Community Structure and Arsenic Biogeochemistry in an Acid Vapor-Formed Spring in Tengchong Geothermal Area, China
Source: PLoS One. 2016 Jan 13;11(1):e0146331. doi: 10.1371/journal.pone.0146331 (PMC4711897; doi:10.1371/journal.pone.0146331)

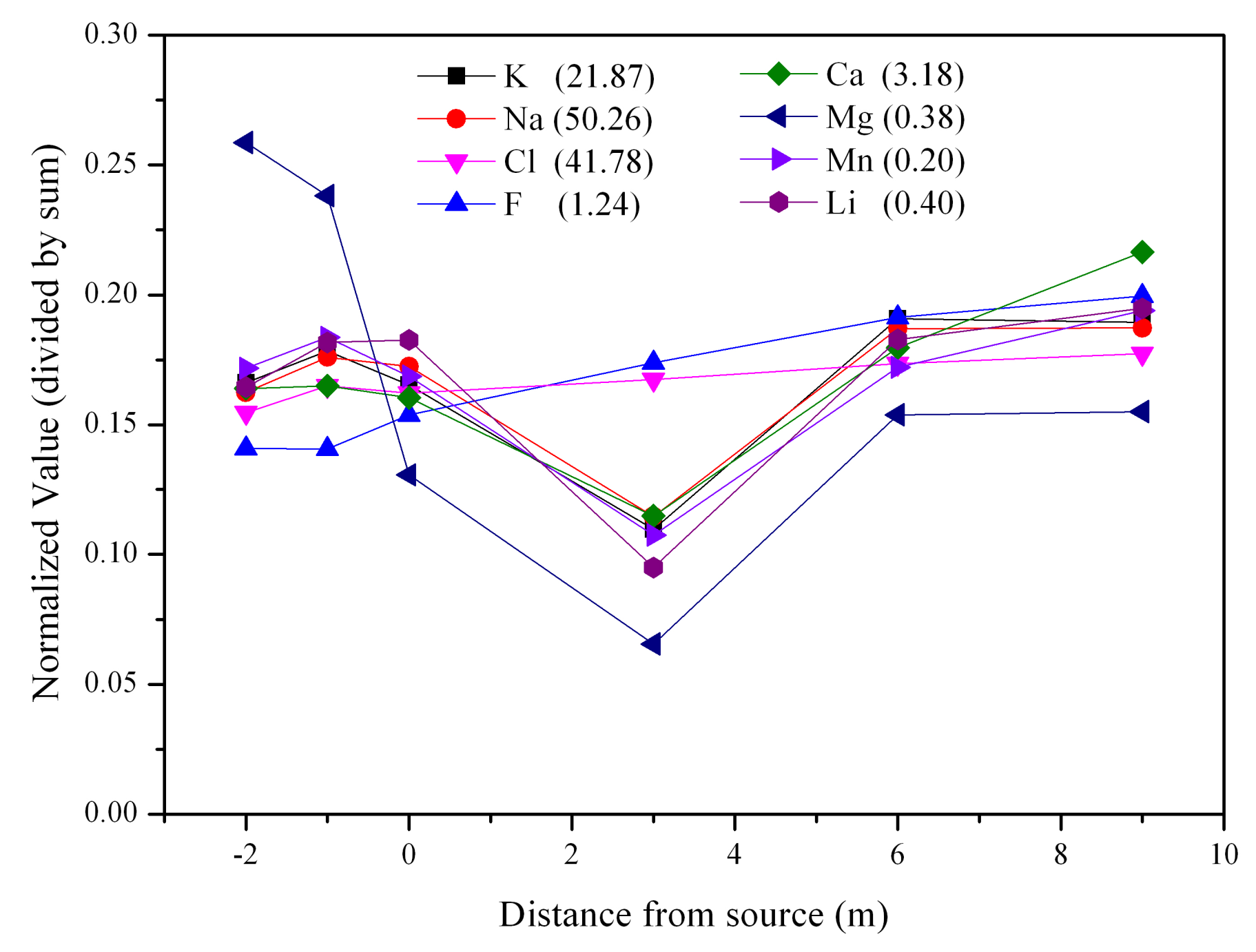

Supplement: S1 Fig — The values in parenthesis were averaged concentrations of ions with a unit of mg/L. (TIF) [file pone.0146331.s006.tif]

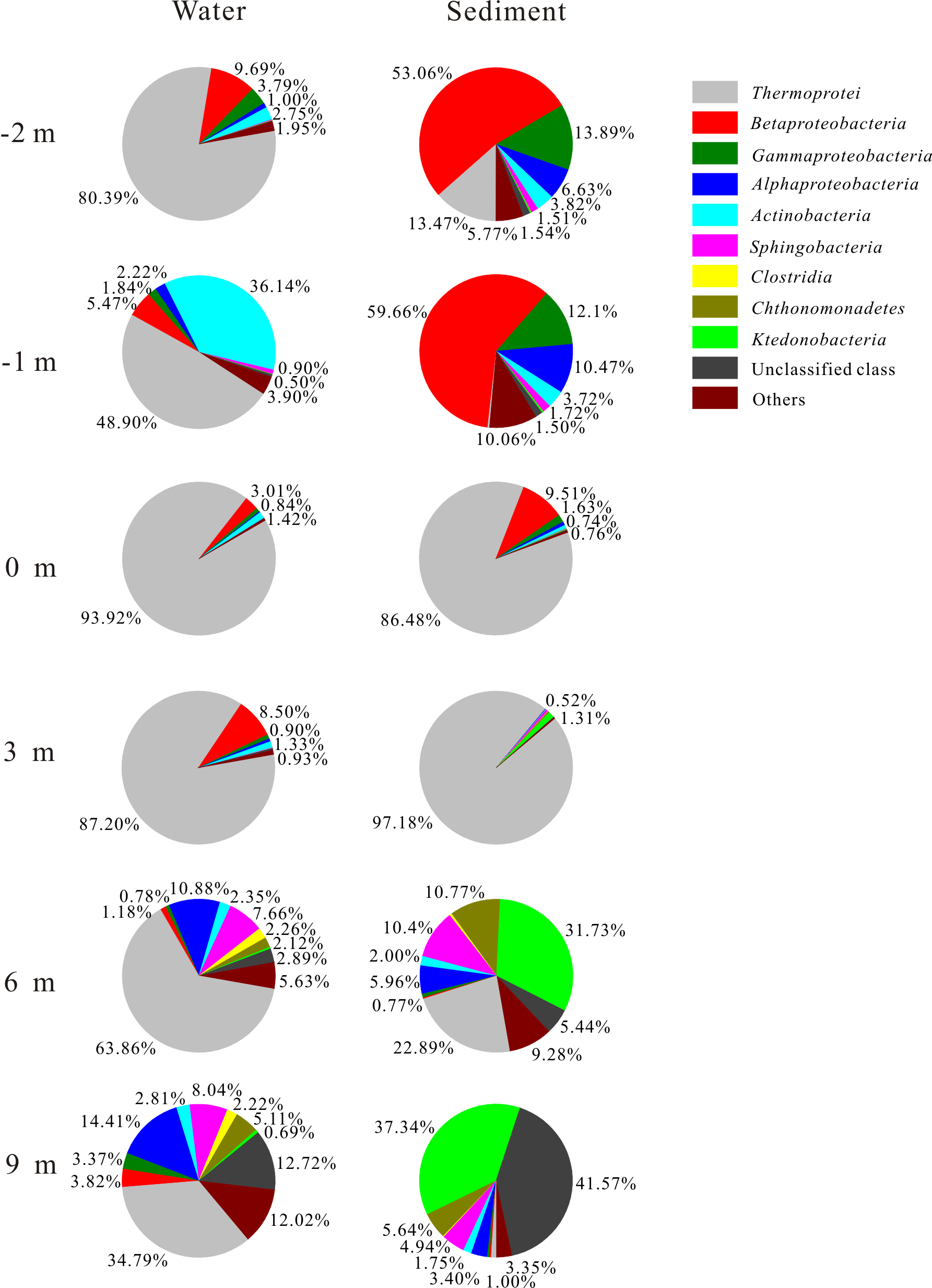

Supplement: S2 Fig — The ratios which exceeded 0.5% were displayed in this figure. (TIF) [file pone.0146331.s007.tif]

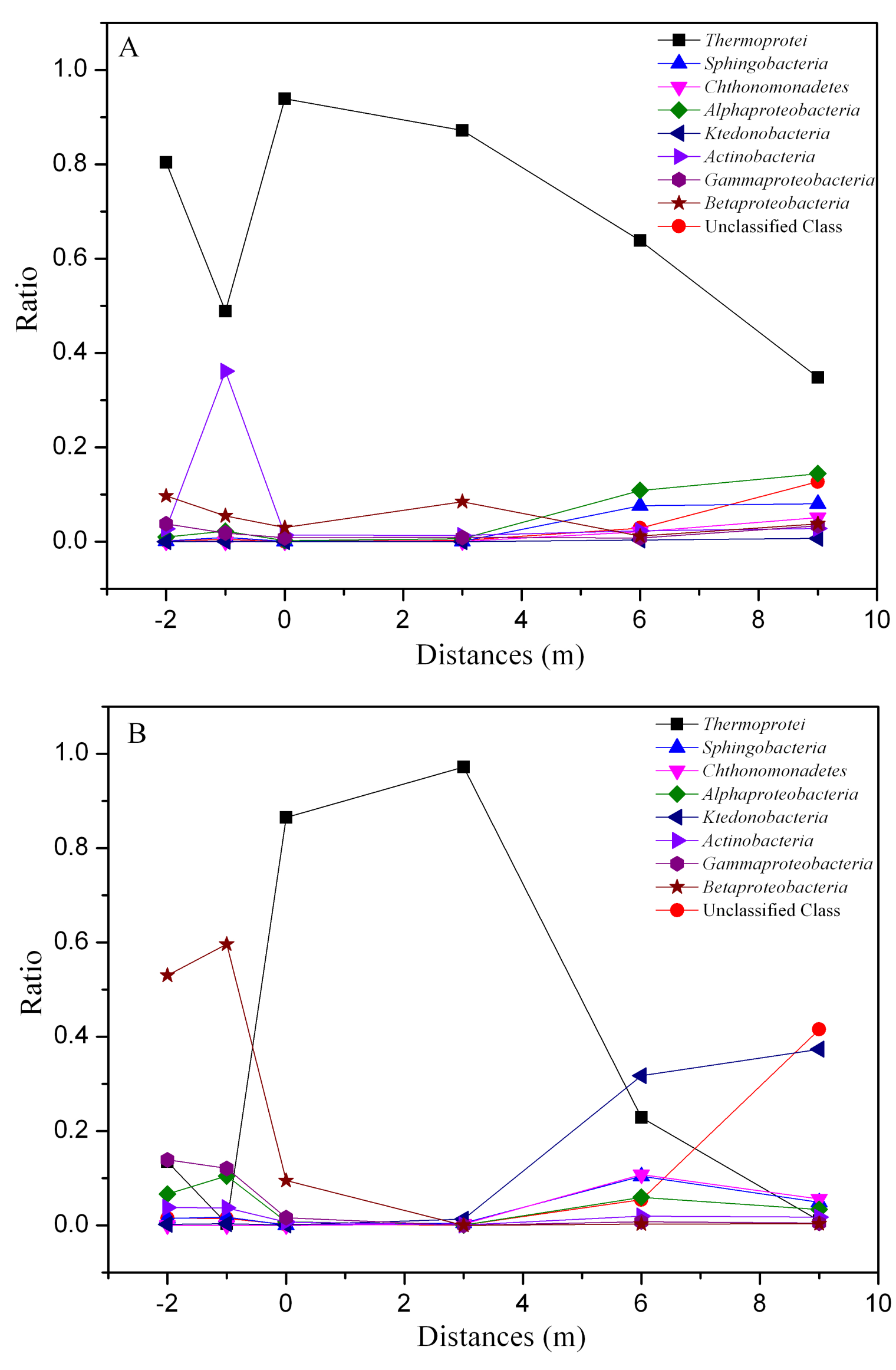

Supplement: S3 Fig — (TIF) [file pone.0146331.s008.tif]
